# Saudi Arabia Household Awareness and Knowledge of Food Safety

**DOI:** 10.3390/foods11070935

**Published:** 2022-03-24

**Authors:** Amira A. Ayad, Nisreen M. Abdulsalam, Najla A. Khateeb, Maha A. Hijazi, Leonard L. Williams

**Affiliations:** 1 Center for Excellence in Post-Harvest Technologies, College of Agriculture & Environmental Sciences, North Carolina A&T State University, 500 Laureate Way, Kannapolis, NC 28081, USA; llw@ncat.edu; 2 Department of Food and Nutrition, Faculty of Human Sciences and Design, King Abdul Aziz University, P.O. Box 42807, Jeddah 21551, Saudi Arabia; nabdulsalam@kau.edu.sa (N.M.A.); mhijazi@kau.edu.sa (M.A.H.); 3 Clinical Nutrition Department, College of Applied Medical Sciences, King Saud bin Abdulaziz University for Health Sciences, P.O. Box 2477, Al Ahsa 31982, Saudi Arabia; khateebn@ksau-hs.edu.sa

**Keywords:** food safety knowledge, food safety practices, Saudi Arabia’s households

## Abstract

Food safety is a critical problem that impacts everyone worldwide. Many countries around the world are becoming increasingly reliant on the availability and safety of their food supply. Despite growing public consciousness about food-related dangers, the growth in food poisoning cases implies that individuals continue to make food consumption, food storage, and food preparation decisions that are less than optimal from a health and safety standpoint. The aim of this study was to assess Saudi Arabia’s households’ knowledge and practices of food safety. A cross-sectional study was conducted to assess knowledge of food safety and practices among a group of households in Saudi Arabia. An online questionnaire using social media (Facebook, Twitter, Instagram, and WhatsApp), and emails communications were distributed. A total of 309 adults (age range 18–59 years) participated in the study. In general, there were no differences between regions regarding the knowledge of food safety. Additionally, most of the participants had good practices of food safety. The findings of our study show that the gender, age, and educational level are factors that have an impact on the knowledge of food safety among respondents. Regarding food safety practices, in general, the results show that the participants had good practices. Therefore, organizations should focus on educational programs, training, holding workshops and activities in public places such as malls, schools, and home visit to improve and increases food safety knowledge and practices.

## 1. Introduction

Globally, food safety is a vital topic that impacts everyone. Many countries throughout the world are becoming completely reliant on the availability and safety of their food supplies [1]. Food-borne diseases (FBD) are a result of the consumption of food that is contaminated with pathogens such as bacteria, parasites, or viruses. There are three major types of hazards; biological hazards are living organisms (including viruses, bacteria, protozoa, molds and parasites), chemical hazards can be artificial chemicals produced by industry or natural chemicals, and physical hazards include stones and fragments of metal or glass [2,3,4]. Food-borne diseases continue to be a major concern worldwide despite improvements in food regulations and food handling that are helping to reduce the incidence of some pathogens in foods [5]. Although states and federal governments regulate food safety practices in commercial food production and food service establishments, there are no guidelines on how to control food preparation and handling in the home [6].

The World Health Organization (WHO) estimated that 33 million years of healthy lives are lost due to eating unsafe food globally each year [7]. European Food Safety Authority (EFSA) reports 5648 (1.1/100,000) food-borne outbreaks affecting 69,553 people, with the household/domestic kitchen as the second most commonly (32.7%) reported setting for outbreaks [8]. To reduce these numbers, several countries and confederations, such as the European Union [9], the USA [10], China [11,12], and most of the Arab Gulf Cooperation Council (GCC) countries [13,14,15], including Saudi Arabia [16] have focused to restructure their food safety systems in order to increase their effectiveness and efficiency, as well as to restore public trust in food safety. Therefore, The United Nations’ Food and Agriculture Organization (FAO) and the World Health Organization (WHO) have collaborated to help food safety authorities assess and alter their national regulatory frameworks to meet national and international demands [17,18]. Although food safety awareness remains a major issue among consumers in both developed and developing countries, developing countries are more likely to suffer from foodborne illnesses because they have poor living standards, poor hygiene, and limited access to health care [19]. The most typical variables associated with reported outbreaks of food-borne illness in households were contaminated raw foods, poor cooking, and intake of food from an unsafe source [20,21].

Accordingly, food safety is defined as a type of assurance that food will not cause illness or harm to the consumer when prepared, handled, and consumed as directed. Food-borne infections are caused by consuming contaminated meals and goods. Food contamination at every level, from manufacture to consumption, results in the growth of bacteria, viruses, parasites, chemical agents, and toxins, which finally cause food-borne illnesses [4,21]. As a result, people all over the world are increasingly concerned about food safety; food production should be done in a safe manner to maximize public health advantages and environmental benefits. The most at-risk groups that are affected by food-borne diseases are pregnant women [22,23], elderly, immunosuppressed people, and children under five years because of their low weight and incomplete development of their immune system [24]. Traditional food safety practices problems could be created at home [25,26], catering and restaurants and industrial processing [27]. Therefore, the aim of this study was to assess Saudi Arabia’s households’ knowledge and practices of food safety.

## 2. Materials and Methods

### 2.1. Study Design and Participants

The design of the study was obtained by following the protocol as per Low, et al. [28] with slight modifications and uploaded to the Google forms platform. The designed questionnaire (which was initially written in English) was translated into Arabic for distribution to Saudi families and then back translated into English. The survey was tested and validated by experts in the food and nutrition department to ensure that the questions were appropriate and avoided any misunderstanding while answering the questions. An online questionnaire was distributed via social media (Facebook, Twitter, Instagram, and WhatsApp), and emails communications. The survey did not require approval by the ethics committee because of the anonymous nature of the online survey and impossibility of tracking sensitive personal data. Initially, we received 422 responses. After eliminating responses that met the exclusion criteria, such as incomplete surveys, out of the age range, and lack of any data, we had 309 responses.

### 2.2. Data Collection

The first section demonstrating demographic data included six questions about gender, age, marital status, employment status, education level, and place of residence to determine the basic information of the households. The second section included food safety knowledge that contained twelve questions (7–19). The final section included food safety practices (20–26), which consisted of seven questions relating to cleaning, hygiene, preparation, and storage habits of household especially in the kitchen.

### 2.3. Statistical Analysis

The collected data were analyzed with Microsoft Excel and the Statistical Package for the Social Sciences version 16.1 [29]. Data on respondents’ responses to terms of study were analyzed using frequency (n) and percentages (%) while the data on the differences between variables were analyzed using cross-tabulations. Values of *p* < 0.05 were considered statistically significant.

## 3. Results

### 3.1. Sociodemographic Characteristics

Data on age, gender, marital status, education level, place of residence, and employment status were collected. Table 1 describes the sociodemographic characteristics of the study population. Over half of the study population (93.5%) were female, 58.3% were between 19 and 29 years old, 22.3% were between 30 and 39 years old, and 3.2% were older than 50 years old. Half of the study population (51.8%) were unmarried, and more than half of the participants were undergraduate students (55.7%). Moreover, about 71.8% of participants were living in the western district.

### 3.2. Knowledge of Food Safety

Five major questions that covered key food safety concepts including the symptoms of food-borne diseases, the physical hazards that can cause food contamination, the bacteria carried by poultry and cause food poisoning, the correct temperature for the refrigerator, and the freezer was presented in Table 2. Most of the participants (90.9%) were aware of the common symptoms of food-borne diseases. However, only half of the participants (57.3–57.9%) were well-informed about the physical hazards that can cause food contamination, and the type of bacteria causing food poisoning that are carried by poultry. Additionally, only (29.1 and 29.4%) of the participants were aware of the difference between the temperature for the refrigerator, and the freezer. Furthermore, our finding showed that the participants with master’s and Ph.Ds. degrees (85.7%, 100, *p* > 0.05), respectively, were significantly more aware of the food safety practices and food handling than the participants with a lower education level, see Table 3. Most of the respondents were between the Eastern region and the Middle region, followed by the Southern region and the Western region, see Table 4. Several published studies have revealed poor consumer knowledge with respect to food hygiene regarding food preparation and storage temperature [30,31].

### 3.3. Practices of Food Safety

There is a remarkably high percentage of female participants (289, 93.5%) compared to male participants (20, 6.5%) for the following questions: food contact surface should be cleaned using sanitizing agents, and bacteria and viruses are microbiological hazards that can cause food-borne illness when transferred to food. Overall, according to our findings, female participants scored higher on all forms of food safety awareness. Therefore, female participants were significantly more knowledgeable on food handling practice than male participants, see Table 5. However, only 26.3% of men and 31.1% of women are aware that tasting or smelling food does not determine whether it is safe to consume. Moreover, 42.1% of the male participants and 30.8% of the female participants reported knowing that the chicken sink drain needed to be cleaned more frequently than weekly to prevent food poisoning. Additionally, our survey results reveal that the majority of survey participants responded positively to the food safety practice questions (20–26) see Table 6. However, from the two questions regarding reheating food that has been cooked at 70 °C and above and thawing frozen food at room temperature and temperatures above, and frozen food not being able to be thawed at room temperature, many participants did not understand these concepts. Only 17.0% of females and 15.8% of males agree that it is hazardous to thaw frozen food at room temperature, as this allows bacteria to grow.

## 4. Discussion

Food-borne illness outbreaks pose serious public health concerns that require immediate action. Food-related illnesses occur for a variety of reasons, including poor food preparation practices that allow pathogens (e.g., natural toxins, poisonous substances, bare-hand contact with food) and other hazards to get into food [32]. Therefore, a lack of hygiene at all stages of food processing, preparation, and serving increases food-borne illnesses [33,34].

Our present study revealed that a total of (309) questionnaires were completed by the household in different regions of Saudi Arabia. Most of the questionnaires (93.5%) were filled out by females and the participants’ average age was 19 to 29 years of age, which is not surprising since most questionnaires were distributed among female-dominated classes at the university. Similarly, our results were consistent with results from [35,36], which showed that the women who participated in the study had a good understanding and application of food safety practices. Additionally, our results are inversely similar with the study of [37], as the majority of the participants (53.4%) were males of 12 to 25 years of age since the surveys were conducted among male-dominated classes at King Saud University. The educational level was generally high with 55.7% of participants holding a bachelor’s degree or higher.

The results of our statistical analysis of the total responses to the food safety (7–11) questions indicate that the majority of participants (90.9, 57.9 and 57.3%) answered correctly about symptoms of food-borne illness, physical dangers of food contamination, bacteria carried by poultry, and the cause of food poisoning as salmonella, respectively.

However, only 29.1% and 29.4% of the participants, respectively, correctly answered the questions regarding refrigeration and freezing temperatures, which is in line with other studies [38,39]. Since two-thirds of the respondents in our study were women, our results suggest that Saudi women still manage food shopping and food preparation more so than men. Furthermore, we identified three age groups, young adults (19–29 years), adults (30–49 years), and seniors (50 years) with 58.3, 34.6 and 3.2%, respectively, indicating that young adults are beginning to acquire knowledge about food safety and preparation. Therefore, our findings agree with the previously reported results of [40] wherein young adults (≤25 years), adults (26–59 years) with 35.7 and 50.7%, respectively, were becoming more involved in food preparation than seniors with 13.7%. Many studies reported that high education could increase the knowledge on food safety [25,33,35,38,41]. Furthermore, our findings support that of Farahat et al.’s 2015 study which concluded that education level influences the likelihood of scoring higher on correct answers. Thus, participants with higher educational levels were more knowledgeable about safe food handling and practices [42]. Comparatively, there was no significant difference observed between education levels and food handling practices of Konya, Turkey consumers [21]. Several studies showed that food-borne illnesses are increasingly linked to unsafe domestic food safety practices [38,41]. Additionally, our results show that there are no significant differences between regions regarding the knowledge of food safety.

According to our survey results, the food safety practice questions (20–26) reveal that the majority of the participants had good practices. Although there were two questions, reheating food cooked above 70 °C does not need to be done, and frozen food cannot be thawed at room temperature, it seems that most participants did not understand these concepts. As an example, only 17.0% of females and 15.8% of males agree that frozen food cannot be thawed at room temperature, which causes the growth of pathogenic bacteria. Our findings agreed with the results from Naeem et al., 2018, which stated that 89.1% of respondents were unaware of the consequences of leaving food at room temperature for >4 h, which may lead to pathogenic bacteria growing to the point where it could cause food poisoning upon consumption [39]. Additionally, several studies indicate that training is vital to improving knowledge and practices related to food safety, and perhaps equal to factors such as demographics and education levels [26,32,33,43].

## 5. Conclusions

This study evaluated knowledge and practices of food safety of households in Saudi Arabia. The findings of our study show that the gender, age, and educational level are factors that have an impact on the knowledge of food safety among respondents. Overall, based on the responses to the questionnaire, the results indicate that the participants had good food safety practices. In conclusion, we recommend that government organizations increase food safety education programs because they have a high impact in increasing awareness among the public. Generally, we suggest that health organizations should aim to improve and increase food safety knowledge and practices by focusing on educational programs, training, workshops and activities held in public locations such as malls, schools, and universities [44].

### Limitation

There are some limitations to this study, for instance, the data did not represent all regions of Saudi Arabia due to the lack of time. For better results, this study should be done considering a longer time frame to cover all regions. In addition, unequal distribution of sample characteristics such as age, gender and educational level affected the outcomes. To fix this problem, select specific participants before distribution. Finally, answering all the survey questions was not required, which give a chance to participants to omit some questions. These situations force the researcher to delete the participants from the statistical analysis. The option of not omitting any survey question should be considered in any further research.

## Figures and Tables

**Table 1:** Respondent demographic characteristics (*n* = 309).

Measure	Frequency (*n*)	Percentage (%)
Gender		
Female	289	93.5 *
Male	20	6.5
Age (years)		
18 and under	12	3.9
19–29	180	58.3 *
30–39	69	22.3
40–49	38	12.3
50 and over	10	3.2
Marital Status		
Married	138	44.7
Divorced	7	2.3
Separated	3	1.0
Widowed	1	0.3
Unmarried	160	51.8 *
The employment status		
Full-time employment	58	18.8
Part-time employment	7	2.3
Unemployed	36	11.7
Self-employed	5	1.6
Homemaker	54	17.5
Student	140	45.3 *
Retired	9	2.9
Education level		
High school	57	18.4
Some college	61	19.7
Bachelor’s degree	172	55.7 *
Master’s degree	14	4.5
Doctoral degree	2	0.6
Professional degree	3	1.0
Region of country		
Middle Region	29	9.4
Northern Region	9	2.9
Eastern Region	8	2.6
Western Region	222	71.8 *
South Region	41	13.3

* Indicates to the highest percentage.

**Table 2:** Correct responses to common food sources of food-borne disease pathogens scale questions among Saudi household.

Questions	Frequency (*n*)	Percentage (%)
Symptoms of food-borne diseases are?		
Fever	1	0.3
Abdominal pain	14	4.5
Vomiting	6	1.9
Diarrhea	7	2.3
All the above	281	90.9 *
Physical hazards that can cause food contamination are?		
Hair	8	2.6
Glass	8	2.6
Metal fragment	66	21.4
Nails	48	15.5
All the above	179	57.9 *
The bacteria that carried by poultry and cause food poisoning is?		
*Salmonella*	177	57.3 *
*E-coli*	40	12.9
*Bacillus*	22	7.1
*Vibrio*	13	4.2
None of the above	57	18.4
What is the correct temperature for the refrigerator		
0–5 °C	90	29.1 *
6–7 °C	64	20.7
8–10 °C	76	24.6
11–13 °C	37	12.0
14–16 °C	42	13.6
What is the correct temperature for the freezer		
0–5 °C	69	22.3
−6–8 °C	47	15.2
−9–12 °C	58	18.8
−13–15 °C	44	14.2
−16–18 °C	91	29.4 *

* Indicates to the highest percentage.

**Table 3:** Respondents’ food safety knowledge based on the education level.

Questions	High School (*n* = 57)	Some College (*n* = 61)	Bachelor’s Degree (*n* = 172)	Master’s Degree (*n* = 14)	Doctoral Degree (*n* = 2)	Professional Degree (*n* = 3)
Symptoms of food-borne diseases are?						
Fever	1.8	0	0	0	0	0
Abdominal pain	10.5	0	3.5	7.1	0	33.3
Vomiting	1.8	1.6	2.3	0	0	0
Diarrhea	1.8	1.6	2.9	0	0	0
All the above	84.2	96.7	91.3	92.9	100	66.7
Physical hazards that can cause food contamination are?						
Hair	3.5	1.6	2.9	0	0	0
Glass	3.5	0	2.9	0	0	33.3
Metal fragment	15.8	21.3	24.4	7.1	50	0
Nails	14.0	18.0	16.3	0	0	33.3
All the above	63.2	59.0	53.5	92.9	50	33.3
The bacteria that carried by poultry and cause food poisoning is?						
*Salmonella*	45.6	36.1	66.3	85.7	100	33.3
*E-coli*	14.0	26.2	8.1	14.3	0	0
*Bacillus*	5.3	6.6	8.1	0	0	33.3
*Vibrio*	8.8	6.6	2.3	0	0	0
None of the above	26.3	24.6	15.1	0	0	33.3
What is the correct temperature for the refrigerator						
0–5 °C	22.8	34.4	30.8	21.4	0	0
6–7 °C	21.1	21.3	20.9	21.4	0	0
8–10 °C	33.3	23.0	22.1	7.1	100	66.7
11–13 °C	8.8	14.8	12.2	14.3	0	0
14–16 °C	14.0	6.6	14.0	35.7	0	33.3
What is the correct temperature for the freezer						
0–5 °C	19.3	19.7	24.4	21.4	0	33.3
−6–8 °C	12.3	18.0	14.0	28.6	50	0
−9–12 °C	19.3	23.0	18.6	0	50	0
−13–15 °C	24.6	13.1	11.6	7.1	0	33.3
−16–18 °C	24.6	26.2	31.4	42.9	0	33.3

**Table 4:** Participant’s food safety knowledge based on the region residence.

Questions	Middle Region (*n* = 29)	Northern Region (*n* = 9)	Southern Region (*n* = 41)	Eastern Region (*n* = 8)	Western Region (*n* = 222)
Symptoms of food-borne diseases are?					
Fever	0	0	0	0	0.5
Abdominal pain	3.4	22.2	2.4	0	4.5
Vomiting	3.4	11.1	2.4	0	1.4
Diarrhea	3.4	0	0	0	2.7
All the above	89.7	66.7	95.1	100 *	91.0
Physical hazards that can cause food contamination are?					
Hair	3.4	11.1	2.4	0.0	2.3
Glass	0	11.1	4.9	0.0	2.3
Metal fragment	13.8	33.3	24.4	25.0	21.2
Nails	6.9	11.1	17.1	12.5	16.7
All the above	75.9 *	33.3	51.2	62.5	57.7
The bacteria that carried by poultry and cause food poisoning is?					
*Salmonella*	72.4 *	44.4	56.1	25.0	57.2
*E-coli*	3.4	22.2	12.2	12.5	14.0
*Bacillus*	3.4	11.1	7.3	12.5	7.2
*Vibrio*	6.9	0.0	9.8	0	3.2
None of the above	13.8	22.2	14.6	50.0	18.5
What is the correct temperature for the refrigerator					
0–5 °C	31.0	11.1	22.0	37.5	30.6
6–7 °C	20.7	44.4 *	17.1	12.5	20.7
8–10 °C	27.6	22.2	36.6	0	23.0
11–13 °C	13.8	11.1	7.3	37.5	11.7
14–16 °C	6.9	11.1	17.1	12.5	14.0
What is the correct temperature for the freezer					
0–−5 °C	10.3	22.2	19.5	50.0 *	23.4
−6–−8 °C	20.7	22.2	14.6	12.5	14.4
−9–−12 °C	13.8	33.3	17.1	25.0	18.9
−13–−15 °C	20.7	11.1	7.3	0	15.3
−16–−18 °C	34.5	11.1	41.5	12.5	27.9

* Indicates to the highest percentage.

**Table 5:** Responses to statements concerning food safety awareness.

	Male						Female						*p* Value
	Right		Wrong		I Do Not Know		Right		Wrong		I Do Not Know		
	*n*	%	*n*	%	*n*	%	*n*	%	*n*	%	*n*	%	
Q12. Good personal hygiene of household will ensure food safety	19	100%	0	0	0	0	278	96.2 *	7	2.4	4	1.4	0.005 ^a^
Q13. To avoid food poisoning, the chicken sink drain should be cleaned every week	8	42.1	8	42.1	3	15.8	185	64.0	89	30.8	15	5.2	0.02 ^a^
Q14. To determine the safety of food, you should taste and smell the food before eating it	13	68.4	5	26.3	1	5.3	183	63.3 *	90	31.1	16	5.5	0.03 ^a^
Q15. To determine the safety of food, check the expiry date before eating it	19	100	0	0	0	0	286	98.96 *	3	1.04	0	0.00	0.005 ^a^
Q16. Cleaning agents are Chemical hazards that can cause food contamination	16	84.2	2	10.5	1	5.3	227	78.5	31	10.7	31	10.7	0.03 ^a^
Q17. Thorough washing of vegetables and fruits in tap water is necessary to prevent food poisoning	17	89.5	2	10.5	0	0	254	87.9	24	8.3	11	3.8	0.03 ^a^
Q18. Food contact surface should be cleaned using sanitizing agents	13	68.4	5	26.3	1	5.3	257	88.9 *	26	9.0	6	2.1	0.09 ^a^
Q19. Bacteria and viruses are microbiological hazards that can cause food-borne illness when transferred to food	16	84.2	1	5.3	2	10.5	261	90.3 *	6	2.1	22	7.6	0.0006

^a^ Kruskal-Wallis Test. * Indicates to the highest percentage.

**Table 6:** Responses to statements concerning food safety practices.

Knowledge of Food Handling	Strongly Disagree		Disagree		Neutral		Agree		Strongly Agree		*p* Value
	F	M	F	M	F	M	F	M	F	M	
Keeping the cooking utensils clean after use is essential	7.6	15.8	3.1	0	6.6	5.3	31.5	15.8	51.2 *	63.2	0.003 ^a^
Raw food should be kept separately from the cooked food	8.3	15.8	4.5	10.5	9.0	0	37.0	21.1	41.2 *	52.6 *	0.006 ^a^
Hand washing with running water is not enough to remove bacteria before touching food	9.7	21.1	10.0	10.5	14.2	15.8	35.6*	21.1	30.4	31.6 *	0.006 ^a^
Avoid bare hand contact with ready to eat food	13.1	26.3	19.7	21.1	23.2	26.3	28.7 *	10.5	15.2	15.8	0.01 ^a^
Food that has been cooked to 70 °C or higher does not need to be reheated	15.6	15.8	30.8 *	42.1 *	31.5	21.1	18.0	15.8	4.2	5.3	0.004 ^a^
Before preparing food, household members should wash their hands with soap and water	9.0	21.1	3.5	0	7.3	0	32.2	21.1	48.1 *	57.9 *	0.004 ^a^
A frozen food cannot be thawed at room temperature	23.9	21.1	27.3*	26.3*	22.1	15.8	17.0	15.8	9.7	21.1	0.1 ^a^

^a^ Kruskal-Wallis Test. * Indicates to the highest percentage. F = Female, M = Male.

## Data Availability

Data is contained within the article.
